# Association between Exposure to *Leptospira* spp. and Abortion in Mares in Croatia

**DOI:** 10.3390/microorganisms12061039

**Published:** 2024-05-21

**Authors:** Iva Zečević, Mathieu Picardeau, Silvijo Vince, Suzana Hađina, Matko Perharić, Zrinka Štritof, Vladimir Stevanović, Iva Benvin, Nenad Turk, Ivana Lohman Janković, Josipa Habuš

**Affiliations:** 1 Department of Microbiology and Infectious Diseases with Clinic, Faculty of Veterinary Medicine, University of Zagreb, 10000 Zagreb, Croatia; hadjina@vef.unizg.hr (S.H.); mperharic@vef.unizg.hr (M.P.); zstritof@vef.unizg.hr (Z.Š.); vstevanovic@vef.unizg.hr (V.S.); ibenvin@vef.unizg.hr (I.B.); turk@vef.unizg.hr (N.T.);; 2 Biology of Spirochetes Unit, National Reference Center for Leptospirosis, Institut Pasteur, 75015 Paris, France; mathieu.picardeau@pasteur.fr; 3 Reproduction and Obstetrics Clinic, Faculty of Veterinary Medicine, University of Zagreb, 10000 Zagreb, Croatia; svince@vef.unizg.hr; 4 Ministry of Agriculture, Veterinary and Food Safety Directorate, 10000 Zagreb, Croatia

**Keywords:** *Leptospira* spp., equine leptospirosis, abortion, seroprevalence, cgMLST

## Abstract

Leptospirosis is one of the most common zoonotic infections and a major problem in terms of both veterinary medicine and public health. However, the disease is under-recognised and under-diagnosed worldwide, particularly in horses. Clinical leptospirosis in horses is mainly associated with recurrent uveitis (ERU), which has recently been studied more intensively, and reproductive disorders, the epidemiology of which is still relatively poorly understood. To enhance our comprehension of abortions caused by leptospirosis in horses and to identify the causative strains, a serological study was carried out with subsequent molecular characterisation of the isolate obtained. Using the microscopic agglutination test (MAT), serum samples from mares that aborted and foetal fluids (when available) were tested for antibodies against *Leptospira* spp. Furthermore, bacteria isolation from kidney cultures was conducted. Of 97 mare serum samples, 21 (21.64%) tested positive, with Grippotyphosa and Pomona being the most frequently detected serogroups. A significantly higher seroprevalence was found in aborting mares compared to the healthy horse population from the same geographical area, as well as a pronounced seasonal variation. Leptospiral antibodies were not detected in any of the foetal fluids, but isolation was successful in 1 case out of 39 (2.56%). Genotyping by multilocus sequence typing (MLST) and core genome multilocus sequence typing (cgMLST) identified the obtained isolate as *Leptospira kirschneri*, serogroup Pomona, serovar Mozdok. Further surveillance and molecular typing of *Leptospira* strains causing abortion in horses would be invaluable in understanding the prevalence and impact of leptospirosis on equine reproductive health in Europe.

## 1. Introduction

Leptospirosis is an emerging zoonosis in domestic animals, wildlife, and humans with worldwide distribution, caused by various pathogenic serovars of the genus *Leptospira*. The diversity within the genus is the result of the constant adaptation of the bacteria to different hosts in different geographical areas. These hosts are responsible for the maintenance of certain serovars in a given geographical area and represent a source of infection for other susceptible species (incidental hosts) [1].

The clinical signs of leptospirosis in domestic animals are variable, ranging from subclinical infections or mild clinical signs associated with host-adapted serovars (e.g., Canicola in dogs, Bratislava in horses, and Hardjo in cattle) to more severe disease following infection with incidental serovars [2,3]. The significance of leptospirosis in horses remains quite uncertain, and, as it is generally asymptomatic, the disease is of limited interest compared to other domestic species. When clinical signs do occur, they usually manifest as recurrent uveitis and reproductive disorders [3,4,5]. Severe septicaemia associated with anorexia, jaundice and fever, renal and hepatic dysfunction, respiratory distress, and pulmonary haemorrhage are increasingly described but have not yet been adequately studied [6,7]. Given the possibility of losing expensive foals or the consequences that recurrent uveitis can have on valuable horses, the importance of leptospirosis in horses is increasingly recognized [3]. The reproductive disorders associated with *Leptospira* infections in horses are mostly late-term abortions, but embryo losses, stillbirths or neonatal mortality, and the birth of weak foals have also been described [5]. Due to the non-specific clinical signs and the difficulty of isolating *Leptospira*, epidemiological studies and diagnoses are mostly based on serology [8]. The gold-standard serological method for diagnosis is the microscopic agglutination test (MAT). Unfortunately, MAT has some technical limitations. Probably the most important limitations are the need for live cultures and the difficulty in standardising the method as it is user-dependent and cross-reactions between related serovars occur [9]. In addition, this test does not distinguish between a current infection and an exposure. This means that a positive MAT result indicates exposure and not necessarily an active (usually chronic) infection leading to abortion. To overcome these limitations, molecular methods are increasingly used to detect pathogenic leptospires in the placenta or aborted foetus [10]. The main disadvantage of this method is the limited epidemiological resolution. The identification of the serovar that caused the infection is important in understanding the transmission mechanism in the herd and in implementing appropriate prevention and control measures.

Studies on the seroprevalence and isolation of *Leptospira* spp. indicate that horses are susceptible to infection with different serovars, the prevalence of which varies greatly depending on the geographical area but also on the health status of the horses tested. In Europe, the serovars most frequently detected in asymptomatic horses are Bratislava and Icterohaemmorhagiae, while the serovar Grippothyphosa is frequently associated with cases of equine uveitis [11,12]. On the other hand, in North America, the serovar Pomona, type Kennewicki, appears to be responsible for most ERU cases and abortions [4,13,14,15]. *Leptospira* serovars associated with equine abortions have been poorly studied in Europe. In the few available reports, the serovars Bratislava and Pomona are identified as the most common causative agents of leptospiral abortions in horses [16,17]. The clinical manifestation of equine leptospirosis seems to be very similar worldwide, but there are still insufficiently investigated differences in the distribution of serovars and the epidemiology of the disease. This study aimed to assess the seropositivity rates among aborting mares and to identify *Leptospira* strains presumably causing the abortion in Croatia.

## 2. Materials and Methods

### 2.1. Sample Population

As part of the measures ordered by the Veterinary and Food Safety Directorate, Ministry of Agriculture of the Republic of Croatia, in the period from 2017 to 2021, 97 serum samples were collected from aborting mares representing various equine species. These included horses (n = 88) (*Equus caballus*) of different categories, such as warmbloods, coldbloods, and ponies, as well as donkeys (n = 9) (*Equus asinus*), spanning different ages. The samples were sent to the laboratory for leptospires at the Faculty of Veterinary Medicine, University of Zagreb, Croatia. Blood samples from privately owned mares and mares from a state stud farm were collected from various regions across Croatia. Samples such as kidneys (n = 37), blood from the heart (n = 30), and pleural (n = 13) and abdominal (n = 20) cavity effusions from aborted foals were processed in all cases when they were available.

### 2.2. Serology

Serum samples from mares, pleural and abdominal effusions, and blood from the hearts of aborted foetuses (if available) were tested for antibodies against *Leptospira* spp. using the microscopic agglutination test (MAT). The MAT was performed according to a standard procedure [18,19] using an antigen panel consisting of 12 pathogenic *Leptospira* serovars, whose presence in Croatia had been confirmed by previous epidemiological analyses (Table 1). Briefly, cultures of *Leptospira* spp., cultivated in Korthof medium within temperature ranges between 28 and 30 °C, up to 10 days old with a density of 2–4 × 10^8^ bacteria/mL were subjected to reactions with the tested sera and serially diluted in phosphate-buffered solution (PBS). The test was read after 2 to 4 h incubation at 30 °C with a dark-field microscope. Results are expressed as dilution titres of serum, including the added antigen. The samples were classified as positive at a cut-off value of 1:200 for Bratislava and Australis and 1:400 for other serovars tested according to Croatian regulations. The highest serum dilution that showed agglutination of at least 50% of leptospires compared to the negative antigen control was considered as the endpoint titre. The presumptive infectious serogroup was determined based on the highest titre for one or more serovars of the particular serogroup.

### 2.3. Isolation and Typing

The kidney cultures were collected using pre-pulled glass micropipettes inserted through the cortex and medulla. The glass micropipettes were transferred with the sample into liquid Korthof medium, incubated at 28–30 °C and observed under a dark-field microscope for up to 16 weeks. If growth of leptospires was observed in the medium, then the isolates were subcultured weekly until the required density, purity, and mobility for further serological and molecular identification procedures were achieved.

The serogroup of the obtained isolate was determined using MAT and hyperimmune rabbit sera for *Leptospira* strains used in an antigen panel.

A multilocus sequence typing (MLST) 6L scheme analysis as described by Ahmed et al. [20] was used for molecular typing of obtained isolate. Briefly, according to the order *adk*, *icd*A, *lipL*32, *lipL*41, *rrs*2, and *sec*Y, nucleotide sequences for each of the genes were trimmed to the reference sizes, concatenated, and aligned using MEGA 11.0 software. Additional sequences that were included in the alignment were retrieved from the online database available at http://pubmlst.org (accessed on 9 July 2019). According to their allelic profiles, found by assessing the Pubmlst database, specific sequence types (STs) were assigned to the isolates.

Further molecular characterisation was performed via core genome multilocus sequence typing (cgMLST) [21]. Illumina sequencing was performed from extracted genomic DNAs of 68 exponential-phase cultures using a MagNA Pure 96 Instrument (Roche, Meylan, France). Next-generation sequencing (NGS) was performed using Nextera XT DNA Library Preparation kit and the NextSeq 500 sequencing systems (Illumina, San Diego, CA, USA) at the Mutualized Platform for Microbiology (P2M) used for analyses. The generated contig sequences together with the sample metadata are available in BIGSdb hosted at the Institut Pasteur (https://bigsdb.pasteur.fr/leptospira/) (accessed on 15 November 2022). The core genome sequence profile was determined using the BIGSdb database. In total, 545 genes were extracted, concatenated and analysed to determine the sequence type.

### 2.4. Statistical Analyses

The statistical analyses were performed using the SAS 9.4 software package (2002–2012 SAS Institute Inc., Cary, NC, USA). Descriptive statistics were performed using the SAS modules PROC MEANS and PROC FREQ and presented as numbers and percentages. Seroprevalence data in the population of healthy horses used for comparison were extrapolated from a previously published report [22]. The odds ratio between the aborted and healthy groups was analysed via logistic regression using the GENMOD procedure, using the binomial distribution and the “logit link” function. The odds ratio (OR) with a 95% confidence interval (95% CI) of abortions caused by *Leptospira* spp. in horses between years and seasons was analysed using the LOGISTIC procedure. Multivariable logistic regression with stepwise selection was employed to construct the model. A significance level of 0.35 was set for variable entry into the model (SLENTRY), while a significance level of 0.30 was required for a variable to remain in the model (SLSTAY). The frequencies were analysed using the chi-square test (PROC FREQ). Differences were considered statistically significant if *p* < 0.05. The graphs were created at a resolution of 300 dpi using the SGPLOT procedure.

Geographic distribution maps of equine abortion were generated using the online tool at datawrapper.de.

## 3. Results

**Figure 1:**
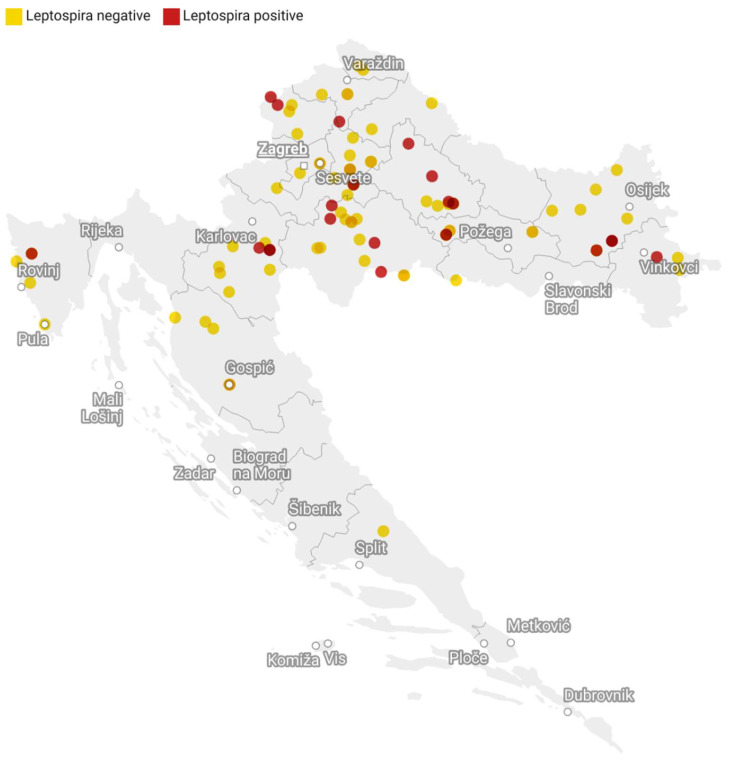
Geographic distribution of tested and *Leptospira*-seropositive cases of equine abortions in Croatia in the period from 2017 to 2021.

During the study period, abortions were reported in 97 equids: 50 coldbloods, 35 warmbloods, three ponies, and nine donkey mares aged between 18 and 348 months. Out of all reported abortions, 42 occurred in winter (43.29%), 28 in spring (28.86%), 22 in autumn (22.68%), and 5 in summer (5.15%). Geographically the dataset was diverse, but most of the abortions originated in the continental part of Croatia (Figure 1).

Of 97 mare serum samples tested via microscopic agglutination (MAT), 21 (21.65%) showed agglutinating antibodies for one or more serovars of *Leptospira* spp. The annual seropositivity rates ranged from 12.5% (2017) to 35.71% (2018), followed by those for the years 2019 (20%), 2020 (23.8%) and 2021 (19.04%), respectively.

The average seropositivity determined in this study was compared with previously determined seropositivity rates among healthy horses from the same population over the same period [22]. The probability of a seropositive *Leptospira* result in mares that aborted was 2.8 times higher than in the healthy population (OR = 2.78; CI 95% = 1.61–4.80, *p* < 0.0002). In some of the years analysed, this difference was even more pronounced. In 2017, the probability of a seropositive result was nine times higher in mares that had an abortion compared to the healthy population (OR = 9.008, CI 95% = 0.87–3.51, *p* < 0.0011). In the other years, the difference was not statistically significant.

**Figure 2:**
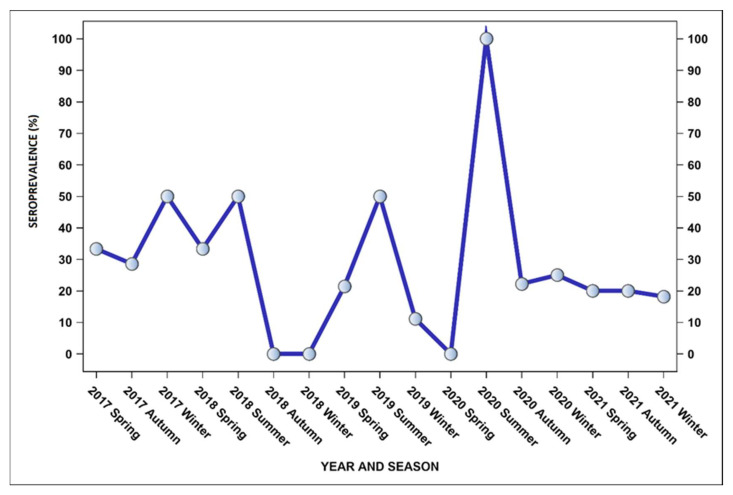
The frequency distribution of *Leptospira*-seropositive mares by season and year of abortion, Croatia, 2017–2021.

Seropositivity within the group of mares that aborted was not significantly influenced by age, category, or geographical origin. The average age of mares was 146.95 months. There was no difference between mares that tested positive for *Leptospira* spp. (M = 142.33; SD = 50.6) compared to the negative group (M = 148.31; SD = 62.62). However, a significant seasonal variation was confirmed, with the highest number of serological positive results found in mares that aborted in summer. The probability of a seropositive *Leptospira* abortion occurring in summer was 11.6 higher than in winter (OR = 11.6; CI 95% = 0.009–0.81, *p* < 0.03), 12.6 higher compared to autumn (OR = 12.665; CI 95% = 1.05–151.84, *p* < 0.04), and 8.9 times higher compared to spring (OR = 8.98; CI 95% = 0.94–85.14, *p* < 0.05). The seropositivity rates of abortions in seropositive mares by season over a 5-year period (from 2017 to 2021) are shown in Figure 2.

**Figure 3:**
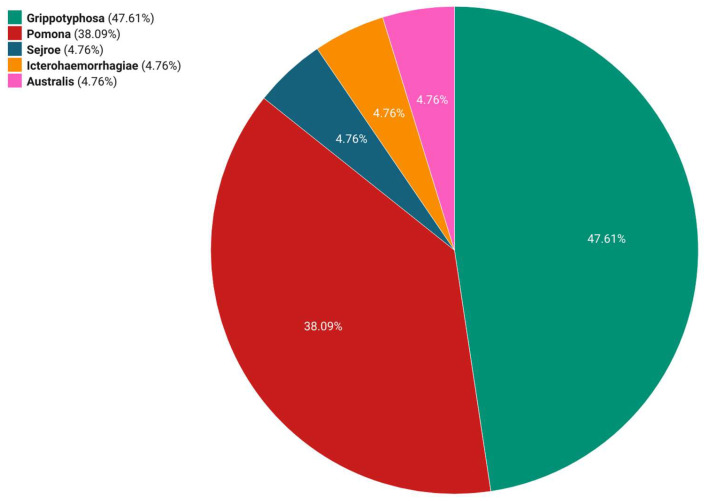
Distribution of *Leptospira* serogroups among seropositive mares that aborted, Croatia, 2017–2021.

**Figure 4:**
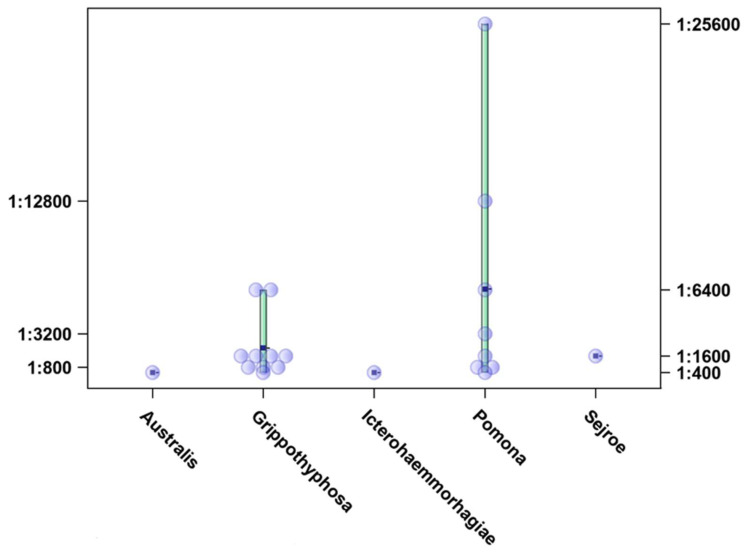
Titre distribution for serogroups detected in seropositive mares, Croatia, 2017–2021. Dots represent the highest titre detected in a tested serum.

During our study period, the most common presumptive infectious serogroups were Grippothyphosa (10/21; 47.61%) and Pomona (8/21; 38.09%). In addition, the serogroups Australis, Sejroe, and Icterohaemorrhagiae were each detected in one sample (1/21; 4.76%) (Figure 3). The titres ranged from 1:400 to 1:25,600, with the highest titre observed for serogroup Pomona (Figure 4). Seasonal variations were observed in the occurrence of the most common presumptive infective serogroup. The most common serogroup in autumn was Pomona (80%), while in winter (57%), spring (50%), and summer (66.66%), Grippotyphosa had the highest prevalence.

None of the heart, abdominal, or pleural effusion samples tested positive in MAT. The isolation of *Leptospira* spp. was successful in one of thirty-seven kidney samples analysed (2.7%). Serological typing with rabbit hyperimmune antisera assigned our isolate to the serogroup Pomona.

Multilocus sequence typing (MLST) results were obtained using the database generated for the 6-locus scheme (https://pubmlst.org/organisms/leptospira-spp) (accessed on 9 July 2019). According to the combination of alleles, this isolate was assigned as sequence type (ST) 98. Strains belonging to this ST correspond to the serogroup Pomona, genomic species *L. kirschneri*, possible serovar: Mozdok or Tsaratsovo.

**Figure 5:**
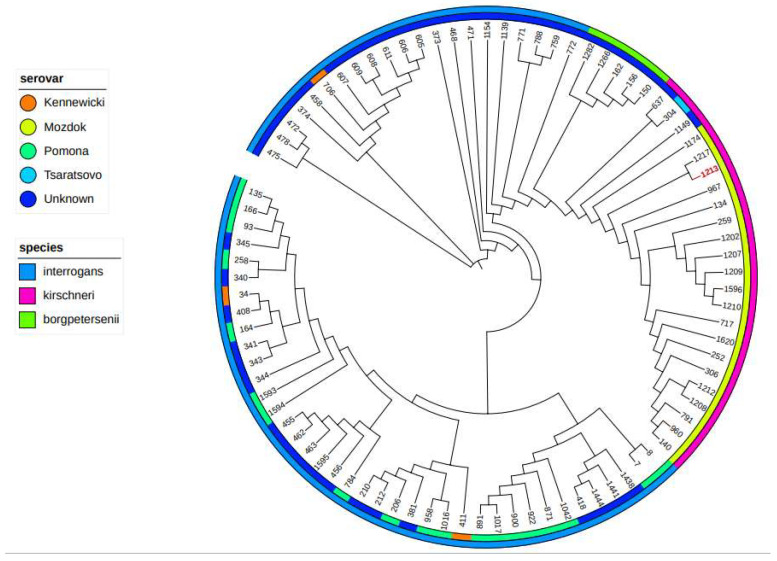
Phylogeny of *Leptospira* strain isolated from a kidney of an aborted equine foetus compared with all isolates of the Pomona serogroup available in the database. Phylogenetic tree based on neighbour-joining method analysis generated and viewed in ITOL. The inner colour represents the serovar (see colour key). The outer circle represents the genomic species (see colour key). The red-coloured isolate ID (1213) represents our isolate clustered with other isolates determined as serovar Mozdok, serogroup Pomona, species *L. kirschneri*. The branch lengths are ignored.

For proceeding the core genome MLST (cgMLST), 545 selected genes were translated, aligned, concatenated, and analysed (Figure 5). According to cgMLST, the clonal group (CG) 73 was established, placing the isolate into the serogroup Pomona, serovar Mozdok, genomic species *L. kirschneri*.

## 4. Discussion

Leptospirosis is endemic in many regions of the world [23]. Although, in domestic animals, it primarily affects livestock and dogs, its impact on equine health and reproduction should not be underestimated. Equine abortions due to *Leptospira* infections pose an economic and welfare challenge for horse owners and veterinarians. In addition, it is important to emphasize that both are at risk of contracting leptospirosis and should take the necessary prophylactic measures to reduce potential exposure during the handling and treatment of animals and biological materials.

Several studies in Europe have reported on the seroprevalence of leptospirosis in horses [22,24,25,26], but these do not focus on abortions, although they emphasise the widespread exposure to different *Leptospira* serovars. To date, conflicting results have been published on the incidence of *Leptospira*-induced abortions in horses and the frequency of the causative serovars in Europe and America. In the USA, leptospirosis, particularly infection with the serovar Pomona, type Kenewicky, is considered an important cause of equine abortion [15]. In Europe, however, leptospirosis as a cause of reproductive disorders in horses is rather poorly researched and is sometimes even regarded as a negligible cause of abortion [27,28]. The results of this study may indicate that equine leptospiral abortion could be of more importance than previously recognized. It is crucial to note that there are currently no available vaccines for leptospirosis for horses in Croatia. Consequently, any antibodies detected with the microscopic agglutination test (MAT) are not vaccine-induced. The seropositive MAT results are not definitive proof that leptospirosis was the cause of the abortion. Nevertheless, we found that the probability of a seropositive result in mares that aborted was significantly higher (2.8 times higher) than in the population of healthy horses in the equivalent years and in the same geographical area. The infection showed a clear seasonality, with the probability of detecting a seropositive reaction being highest in summer. This epidemiological pattern with the highest leptospirosis incidence rates occurring in summer and autumn has already been described in the area [29]. The highest reactivity against the serogroups Grippotyphosa and Pomona was observed throughout the study period. Compared to previous leptospirosis serosurveys in horses in Croatia, a significant increase in the seropositivity rates of Grippothyphosa and a decrease in the serogroup Australis was observed [17]. The titres were also relatively high, with the highest ones observed for the serogroup Pomona.

It has already been suggested that the variation in the prevalence of equine leptospirosis in different geographical areas of Europe may be explained by variations in the number and type of reservoirs hosts [30]. Croatia, with its diverse, water- and forest-rich landscape, is a well-known endemic area of leptospirosis [23,29]. In the studied area, the most probable maintenance hosts and source of the infection for the serovar Grippotyphosa are small rodents (voles and mice). On the other hand, two serovars of the serogroup Pomona were detected in previous epidemiological investigations; the serovar Pomona carried by pigs and the serovar Mozdok carried by the striped field mouse (*Apodemus agrarius*) [1,31].

Research into the epidemiology of leptospiral abortion in horses is hampered by the limitations of diagnostic methods. It is noteworthy that the antigen panel of 12 serovars utilized in this study is grounded on previous epidemiological investigations conducted in Croatia, and that they do cover most of the infected serovars reported in Europe. Nonetheless, it is imperative to consider that horses can be transported across continents; in these cases, they may come across other serovars not encompassed in this panel. In addition, seroreactivity indicates exposure, not necessarily active infection, and molecular methods have a very limited ability to identify the infective serovar, i.e., the indirect source of infection. Molecular typing methods have been developed. Multilocus sequence typing (MLST) is one of the most relevant methods [9,20,32] and most commonly used techniques, even though its design is based on a limited number of genomes and the level of discriminatory power achieved. Each isolate is characterised by the combination of alleles at each locus that form its allelic profile or sequence type (ST). In our study, the molecular approach to a kidney isolate derived from an aborted foal resulted in the strain being assigned to the genomic species *L. kirschneri*, serogroup Pomona, most likely serovar: Mozdok. Given the heterogeneity of *Leptospira*, the conventional MLST approach is not applicable to all clades and species, limiting the current knowledge of the biodiversity and epidemiology of *Leptospira* spp. To achieve an even higher resolution, other approaches have been developed, such as the core genome MLST (cgMLST), which extends MLST concepts to the core genome [21]. To the best of the authors’ knowledge, this study was the first in which cgMLST was performed on an isolate from the kidney of an aborted foal, clustering the isolate with other isolates assigned as genomic species *L. kirschneri*, serogroup Pomona, serovar Mozdok.

Until recently, molecular typing required the isolation of bacteria from clinical material. Isolation procedures are laborious, time-consuming, and often unsuccessful. Recently, however, hybridisation capture followed by Illumina core genome sequencing has been successfully performed directly on clinical material [33]. This particular approach could lead to a better understanding of the distribution of *Leptospira* strains linked to probable hosts and geographical spread.

In conclusion, while specific data on abortions in horses due to *Leptospira* infections in Croatia and Europe are not readily available, the existing research emphasises the importance of vigilance and proactive measures in mitigating the impact of leptospirosis on equine reproductive health.

## Figures and Tables

**Table 1:** Antigen panel used for the screening of horses with suspected leptospirosis.

No.	Serogroup	Serovar	Strain	Genomic Species
**1**	Grippotyphosa	Grippotyphosa	Moskva V	*L. kirschneri*
**2**	Sejroe	Sejroe	M4	*L. borgpetersenii*
**3**	Australis	Australis	Ballico	*L. interrogans*
**4**	Australis	Bratislava	Jež Bratislava	*L. interrogans*
**5**	Pomona	Pomona	Pomona	*L. interrogans*
**6**	Pomona	Mozdok	5621	*L. kirschneri*
**7**	Canicola	Canicola	Hond Utrecht IV	*L. interrogans*
**8**	Icterohaemmorhagiae	Icterohaemmorhagiae	RGA	*L. interrogans*
**9**	Tarassovi	Tarassovi	Peterpelitsin	*L. borgpetersenii*
**10**	Sejroe	Saxkoebing	Mus 24	*L. interrogans*
**11**	Ballum	Ballum	Mus 127	*L. borgpetersenii*
**12**	Batavie	Batavie	Swart	*L. interrogans*

## Data Availability

Data are contained within the article.
